# Investigating the mechanisms by which selective attention affects subsequent preferences and choice

**DOI:** 10.1038/s41598-022-23859-6

**Published:** 2022-11-11

**Authors:** Martin Egger, Arnd Florack

**Affiliations:** 1 Department of Occupational, Economic and Social Psychology, Faculty of Psychology, University of Vienna, Vienna, Austria; 2 Department of Occupational, Economic, and Social Psychology, Faculty of Psychology, University of Vienna, Universitaetsstrasse 7, 1010 Vienna, Austria

**Keywords:** Psychology, Human behaviour

## Abstract

In two experiments, we investigated two untested assumptions regarding the mechanism by which selective attention during search affects subsequent preferences for objects. First, we tested whether an increase in visual competition during search increases preferences for relevant objects and decreases preferences for irrelevant objects subsequent to search. Second, we tested whether searching for objects increases the perceived fluency to process relevant objects and decreases the perceived fluency to process irrelevant objects. Our results show that search can affect relevant and irrelevant objects differently. Selective attention increased preferences for target objects subsequent to search, whereas selective attention did not affect preferences for distractors. Furthermore, our results indicate that searching for a target object increased the perceived fluency for this target object during choice, whereas ignoring a distractor product blocked mere exposure effects. Contrary to assumptions made in previous research, we found no indication that the competition for visual resources during search is linked to preferences for targets or distractors.

## Introduction

Countless times each day, people search for different objects, like the car keys or specific products in a supermarket shelf. During search, selective visual attention enables us to find what we are searching for by focusing on the information important for the task while ignoring the irrelevant or distracting information^1,2^. Previous research has provided evidence that, as a side effect, selective visual attention during search also affects evaluations of objects subsequent to search^3,4^. For example, researchers have observed that when searching for color patterns or faces, irrelevant stimuli have been subsequently rated as less trustworthy or cheerful than control stimuli^5–10^, and the liking for line patterns increased when they were the target of the search^11^. Similarly, in recent studies, products presented next to target products were less preferred subsequent to search^12^, whereas the preferences for the target products increased^12–14^.

Despite the intriguing evidence that selective attention affects preferences, the mechanisms by which searching for an object can affect preferences subsequent to search are not yet fully understood. The literature has discussed a number of different explanations to explain the mechanisms of selective attention on objects subsequent to search^10,12,13,15,16^. According to the currently most accepted explanation, selective attention modulates the neuronal processing of objects exposed in a search task which subsequently affects evaluations of^17,18^ or preferences for these objects^12^. However, the exact mechanism by which neuronal processes during search transfer to subsequent evaluations of these objects is still under debate^10,12,13,15,16^.

Previously, Janiszewski et al.^12^ made three crucial assumptions regarding how selective attention during search affects subsequent preference choices. First, the researchers assumed that search tasks not only modulate the neuronal processing for objects exposed during search, but importantly, that the effects of these modulated neuronal processes carry over to choice situations temporally separated from search. Second, the researchers assumed that a stronger competition for visual processing during search increases the neuronal modulation and therefore increases the effects of search on subsequent preferences. For example, competition during search can increase with an increase in the difficulty to identify the target of a search task against distracting stimuli in the search display. Third, Janiszewski et al.^12^ assumed that the neuronal processes modulated during search affect the perception of objects during subsequent choice, affecting preferences in favor of a former target and to disadvantage a former distractor. Therefore, whereas the first and second assumptions describe the possible mechanism by which effects of search can transfer beyond the search context, the third assumption describes how these mechanisms might happen to influence the preferences during subsequent choice.

Whether the neuronal modulation during search can indeed explain the effects of search on preference choice, when temporally separated from search, needs more thorough investigation. Empirical evidence supporting the assumption that effects originating from search-specific neuronal processes transfer to subsequent preference choice is limited^12^, and the proposed effects on preference choices only partly replicated^13,14,19^. Therefore, the current research had two main objectives. First, we test whether an increase in visual competition during search increases preferences for relevant target objects and decreases preferences for irrelevant distractor objects temporally separated from search. Second, we test whether the exposure of objects during search increases the perceived fluency to process relevant objects and decreases the perceived fluency to process irrelevant objects.

## Theoretical background

According to the biased competition model, selective attention during search serves to select between relevant and irrelevant information during visual information processing^20^. To save processing capacities during visual search, our cognitive system increases the signal-to-noise ratio of important versus irrelevant information. By biasing the neuronal processing during search, the target object becomes easier to differentiate from other objects and consequently easier to identify^21^. On the neuronal level, this bias is accomplished by enhancing synchronicity and selectivity in the neuronal firing rate for the target object^2^. To counteract a higher competition during search, and enhance search performance, the alteration of the neuronal processing during search is amplified as the density of distracting information increases^22^. To further amplify target processing, neuronal responses for distracting information are inhibited during search^23^.

Although search processes can modulate neuronal responses to stimuli during search^1,2^, there is currently insufficient research on whether such a modulation, activated during search, can indeed outlive the search context, and transfer to situations temporally separated from search. At first glance, the empirical results provided by Janiszewski et al.^12^ seem to support the predictions derived from the biased competition model that a higher competition during search increases effects of search on subsequent product preferences in two-alternative search tasks. However, the design of the mentioned study does not exclude an alternative explanation, namely that differences in visual attention spent on the different products produced the reported results. In Janiszewski et al.’s^12^ design, the presentation time during search was limited to one second, and the target and distractor products were presented clearly distinguishable and separated from each other during search. Only the distance *between* the products varied. In such a setting, the visual attention to products separated by larger distances also requires longer saccades when the eyes shift from one product to the other. Consequently, more time is needed for the eye movements and less time remains to actually attend to the target product. If one object receives more attention than another, this can subsequently increase preferences for the longer-attended object^24–26^.

Manipulating the distance *between* two products is also insufficient to induce two distinct conditions of high and low competition on the neuronal level during search. When two products appear next to each other on a white background, but without overlay, each product can still be easily perceived on its own, and large effects of facilitation and inhibition might not be necessary to identify the target during search. In addition, the fovea centralis of the eye, in which visual information is processed most precisely and accurately, only covers up to 3° of visual angle^27^. Therefore, a product placed near another product might not elicit extensive neuronal competition during search, when the receptive fields in the visual cortex do not overlay enough^1,28,29^.

Furthermore, the mechanism by which neuronal effects, induced during search, transfer to subsequent situations is a matter of ongoing debate^10,12,13,15,16,30^. Janiszewski et al.^12^ argued that biased competition during search processes might affect how easily objects can be perceived subsequent to search. Therefore, a possible, but so far untested explanation is that search processes alter not only how easy or difficult objects can be processed during search itself^1^, but also how easy it is to process these objects when encountered subsequent to search. Differences in the processing fluency to perceive two objects, induced during search, could transfer to subsequent situations and serve as an additional informational cue in favor of the easier-to-process object during preference choice^31–33^. Researchers have repeatedly discussed the potential relevance of search-specific processes on processing fluency^9,12,34^. However, researchers so far have not directly investigated whether search tasks can actually induce differences in perceived processing fluency, which persists to a temporally separated choice situation.

## Overview of experiments and hypotheses

In two experiments, participants searched for designated food products in the presence of irrelevant food products. Temporally separated from the search task, participants indicated their preferences for previously exposed food products (Experiment 1), or their perceived fluency to process these products (Experiment 2). We used food products because they offer complex stimulus information and are relevant for daily search tasks such as supermarket shopping.

### Experiment 1

We designed Experiment 1 as a reaction-time experiment to test the assumption that competition for visual processing during search can affect preferences for target and distractor objects. We placed products next to each other, but spatially separated, during search (low competition) as well as partly overlapping during search (high competition). To measure preferences during choice, we used two-alternative forced-choice tasks with novel products as comparison alternatives. Biased competition during search facilitates target processing by increasing the neuronal synchronicity and selectivity for the target stimulus^1^. Importantly, the facilitation to process the target increases with an increase in competition during search in order to support the search process^2^. Therefore, in line with previous research^12^, we expected that higher competition during search, compared to less competition, *increases* effects of biased competition, and therefore higher competition increases the preferences for target products subsequent to search.

H1a: Target products presented in highly competitive search tasks are subsequently preferred more often than target products exposed in less competitive search tasks.

Similarly, we expected that higher competition compared to less competition *decreases* the preferences for distractor products subsequent to search.

H1b: Distractor products presented in highly competitive search tasks are subsequently preferred less often than distractor products exposed in less competitive search tasks.

We either repeated targets or distractors in three consecutive search tasks, or exposed them in only one search task. We expected that a repetition of target or distractor products in multiple search tasks would amplify the effects of competition.

H2a: Target products exposed in multiple consecutive search tasks are subsequently preferred more often than target products exposed in only one search task.

H2b: Distractor products exposed in multiple consecutive search tasks are subsequently preferred less often than distractor products exposed in only one search task.

Additionally, we used the repetition in three consecutive search tasks to test whether an increase in competition during search also increases the processing speed for targets and the inhibition of distractors. The biased competition model predicts that an increase in visual competition amplifies target processing and inhibition for distractors^1,2^. We expected that, between consecutive search tasks with varying distractors, the decrease in response times to identify a target diminishes more strongly in highly competitive search compared to low competitive search.

H3a: The processing speed to identify target products during search increases more strongly when the target is repeated subsequently in highly competitive search tasks, compared to less competitive search tasks.

Since biased competition inhibits distractors during search^1,20^, we expected that repeating only a distracting product during consecutive search tasks would also increase the speed to identify varying target products.

H3b: The processing speed to identify target products during search increases more strongly when the distractor is repeated subsequently in highly competitive search tasks, compared to less competitive search tasks.

### Experiment 2

In Experiment 2, we investigated whether selective attention during search has an effect on the subjective ease to perceive a product during a subsequent choice. We used a procedure already applied in previous research to capture positive effects of target products and negative effects for distractor products, simultaneously controlling for number of exposures and exposure duration^12,13^. Importantly, previous research indicated that the subjective experience of fluency, compared to objective measures such as response times, more accurately predicts the outcome of a decision process^31,35^. Furthermore, people are able to accurately report their subjective experience of fluency^36,37^.

We again applied an exposure phase with two-alternative search tasks in which participants searched for target products while ignoring distractor products. In a subsequent two-alternative forced-choice phase, participants indicated which product they perceived more fluently. We again used novel products as comparison during the two-alternative forced-choice tasks. Furthermore, we included a second comparison alternative during choice to control for mere exposure effects. This second comparison alternative comprised products that we presented equally often and for the same amount of time as target and distractor products, but that were not involved in a search task (termed neutral products). In line with previous research^12,13^, we expected an increase in preferences for target products and a decrease in preferences for distractor products.

H4a: Products that were targets in a search task are subsequently perceived more fluently than novel products, or neutral products not involved in a search task.

H4b: Products that were distractors in a search task are subsequently perceived less fluently than novel products, or neutral products not involved in a search task.

## Results

In Experiment 1, we tested whether competition for visual processing during search affects preferences for targets and distractors subsequent to search. In Experiment 2, we tested whether a search operation can affect the perceived fluency to process target and distractor products subsequent to search. Table 1 provides a summary of our findings.

**Table 1:** Summary of hypotheses and findings.

Hypothesis	Short description		Conclusion
H1a	Higher competition during search increases preferences for targets	×	Not confirmed
H1b	Higher competition during search decreases preferences for distractors	×	Not confirmed
H2a	Repeating targets in subsequent search tasks increases preferences for targets	✔	Confirmed
H2b	Repeating distractors in subsequent search tasks decreases preferences for distractors	×	Not confirmed
H3a	Competition during search increases the processing speed of targets	✔	Confirmed
H3b	Competition during search increases the inhibition of distractors	✔	Confirmed
H4a	Search increases the perceived fluency for targets	×	Not confirmed
H4b	Search decreases the perceived fluency for distractors	×	Not confirmed

### Experiment 1

In a preliminary analysis, we analyzed the percentage of correct target selections in the exposure phase. This revealed that participants, on average, selected the target correctly in 97.7% (*SD* = 2.2) of all search trials, indicating very good search accuracy. To test our hypotheses, we calculated linear mixed-effects models (LMM) and generalized linear mixed-effects models (GLMM). We accounted for the repeated measurements by adding a random intercept for the subject level. We reported estimated marginal means and standard errors on the response scale, as well as bootstrapping confidence intervals for all model results. Furthermore, we calculated one-sample *t*-tests and paired-sample *t*-tests. For all *t*-tests, we calculated the choice percentages as an aggregated mean value of all choice tasks for each participant and the respective combination of competition and repetition. For all *t*-tests, we additionally report the Bayes factor (BF_10_).

For all analyses involving reaction-time responses, we excluded outliers, defined as more than three standard deviations above and below the mean. We calculated the means and standard deviations separately for the two repetition conditions to avoid confounds. We excluded those trials for which participants selected the wrong target location during the exposure phase from all analyses. If a target was selected wrongly in one search task of a repetition trial, we excluded all search tasks of this repetition trial from further analysis.

#### Preference choice percentages

**Figure 1:**
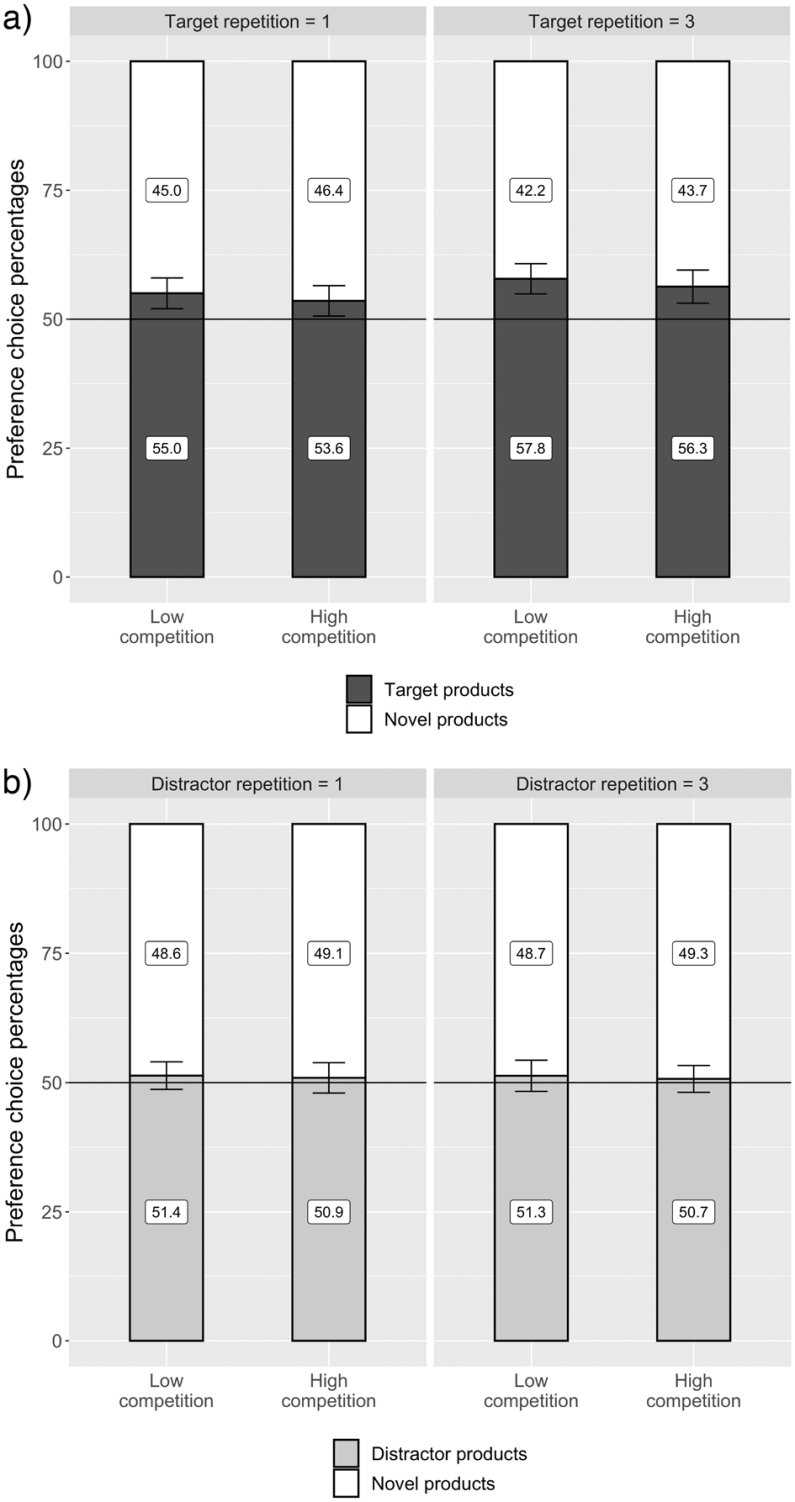
Preference choice percentages (Experiment 1). Preference choice percentages of the two-alternative forced-choice tasks in Experiment 1 for (**a**) target products and (**b**) distractor products. Error bars show 95% confidence intervals.

As a first step, we calculated a paired-sample *t*-test to test whether, across all conditions, target products are preferred more often subsequent to search compared to distractor products. Indeed, target products (*M* = 55.7%, *SD* = 11.1) were preferred more often compared to distractor products (*M* = 50.9%, *SD* = 9.4), *t*(173) = 4.41, *p* < 0.001, *d*_z_ = 0.33, BF_10_ = 807.13. Furthermore, we calculated one-sample *t*-tests analyzing whether the choice percentages for target and distractor products for each combination of competition and repetition differed significantly from choosing randomly in the two-alternative forced-choice tasks (i.e. 50%). For target products, all combinations of competition and repetition resulted in choice percentages significantly different from choosing randomly during choice. However, for the distractors, none of the combinations of competition and repetition differed significantly from choosing randomly during choice (Fig. 1, Table 2).

**Table 2:** Preference choices for target and distractor products (Experiment 1).

Product	Condition		% Chosen		*t (*173)	*p*	*d*	BF_10_
	Competition	Repetition	*M*	*SD*				
Target	low	1	55.0	19.9	3.32	0.001	0.25	16.42
	low	3	57.8	19.6	5.27	<0.001	0.40	> 1000
	high	1	53.6	19.7	2.38	0.018	0.18	1.32
	high	3	56.3	21.4	3.88	< 0.001	0.29	> 100
Distractor	low	1	51.4	17.8	1.00	0.317	0.08	0.14
	low	3	51.3	20.7	0.86	0.390	0.07	0.12
	high	1	50.9	19.6	0.61	0.541	0.05	0.10
	high	3	50.7	17.3	0.54	0.590	0.04	0.10

Results for one-sample *t*-tests against the expected frequency of choosing randomly during the two-alternative forced-choice tasks (i.e. 50%). Values above 50% indicate a higher preference for target or distractor products compared to novel products.

#### Effect of competition on preferences

We expected that target products exposed during a highly competitive search are more often preferred over novel products than target products exposed during less competitive search (H1a). Furthermore, we expected that distractor products exposed during a highly competitive search are less often preferred over novel products than distractor products exposed during less competitive search (H1b). Finally, we expected that repetition of targets and distractors in subsequent search tasks increases the effects on preferences (H2a and H2b). To test our hypotheses, we calculated two GLMM, one for target and one for distractor products, with logit link functions for the binary outcome variable of preference choice (preferred over novel product: “yes” or “no”). We included a fixed factor predictor for competition. To control for a repetition effect between repeating and not repeating search trials, we also included repetition as a fixed factor, as well as the interaction of competition and repetition. We compared the other conditions to the reference category of searching under low competition without repetition. Furthermore, we calculated contrast tests using paired-sample *t*-test for which we provide the Bayes factor.

Our analysis revealed that, for the target products, the interaction effect of competition and repetition predicting preference choice was not significant, *b* = 0.01, *SE* = 0.12, *z* = 0.05, *p* = 0.962, 95% CI [− 0.23, 0.26]. Removing the nonsignificant interaction term from the model revealed that repeating a target during subsequent search tasks increased preferences compared to presenting a target in a single search task, *b* = 0.12, *SE* = 0.06, *z* = 2.03, *p* = 0.042, 95% CI [< 0.01, 0.23], whereas increasing the competition during search did not increase target preferences, *b* = − 0.06, *SE* = 0.06, *z* = 1.08, *p* = 0.279, 95% CI [− 0.18, 0.05]. Additionally, we calculated contrast tests for the competition condition, *t*(173) = 2.08, *p* = 0.039, *d*_z_ = 0.07, BF_10_ = 0.17, as well as for the repetition condition, *t*(173) = 2.08, *p* = 0.039, BF_10_ = 0.62.

For distractor products, the interaction effect of competition and repetition predicting preference choice was also nonsignificant, *b* = − 0.02, *SE* = 0.12, *z* = 0.16, *p* = 0.874, 95% CI [− 0.24, 0.20]. After removing the nonsignificant interaction term from the model, the analysis showed no difference in distractor preferences subsequent to highly competitive search tasks compared to low competitive search, *b* = − 0.02, *SE* = 0.06, *z* = 0.40, *p* = 0.690, 95% CI [− 0.14, 0.09], and no effect of repetition on distractor preferences, *b* = − 0.01, *SE* = 0.06, *z* = 0.20, *p* = 0.842, 95% CI [− 0.14, 0.10]. Additionally, we calculated contrast tests for the competition condition, *t*(173) = 0.38, *p* = 0.701, BF_10_ = 0.13, as well as for the repetition condition, *t*(173) = 0.21, *p* = 0.835, BF_10_ = 0.12.

#### Effect of competition on processing speed

We expected that the speed to identify target and distractor products during highly competitive search increases between subsequent search tasks, compared to less competitive search (H3a and H3b). Importantly, to calculate this gain in processing speed between search tasks, we only included targets and distractors involved in repeating search tasks in this analysis. To test our hypotheses, we calculated two LMM, one for target products and one for distractor products, with the change in processing speed as the outcome variable. We computed the change in processing speed by calculating the average change in response times between the three subsequent search tasks of each repetition trial. In the LMM, we included a fixed factor predictor for competition, whereas low competition was the reference category.

Our results show that, as implied by the biased competition model, repeatedly searching for the same target product under high competition decreased response times to identify the target, compared to low competition, *b* = − 8.89, *SE* = 3.29, *t*(2096.5) = 2.70, *p* = 0.007, 95% CI [− 15.57, − 2.40]. Similarly, repeatedly ignoring the same distractor product under high competition decreased response times to identify the targets more strongly, compared to low competition, *b* = − 13.07, *SE* = 3.81, *t*(2221) = 3.43, *p* < 0.001, 95% CI [− 20.07, − 5.32].

#### Supplementary analyses

We additionally analyzed whether (a) the gain in processing speed during search predicts preferences during choice, and whether (b) the total presentation duration during the response-dependent search tasks predicts preferences based on differences in exposure duration. None of these analyses showed significant effects (see Supplementary Information).

### Experiment 2

In a preliminary analysis, we analyzed the percentage of correct target selections in the exposure phase. This analysis revealed that participant selected the target correctly in 99.5% (*SD* = 1.8) of all search tasks, indicating very good search accuracy. To test our hypotheses, we calculated the choice percentages as an aggregated mean value of the single choices from the two-alternative forced-choice tasks for each participant, separately for target and distractor products and comparison alternatives. We computed paired-sample *t*-tests as well as one-sample *t*-tests against the expected frequency of choosing randomly (i.e. 50%) during the two-alternative forced-choice tasks. For these analyses, we excluded all trials for which the target was not selected correctly during the exposure phase. Additionally, for all *t*-tests, we report the Bayes factor (BF_10_).

We hypothesized that selective attention during the exposure phase would lead to an increase in perceived fluency for target products during choice (H4a) and to a decrease in perceived fluency for distractor products (H4b). As a first step, we analyzed whether search increased the perceived fluency for target products compared to distractor products. For neutral and novel comparison conditions combined, our results indicate that the perceived fluency of target products (*M* = 54.3%, *SD* = 12.7) is indeed higher than the perceived fluency of distractor products (*M* = 48.8%, *SD* = 11.1), *t*(115) = 2.06, *p* < 0.001, *d*_z_ = 0.31, BF_10_ = 44.88.

**Figure 2:**
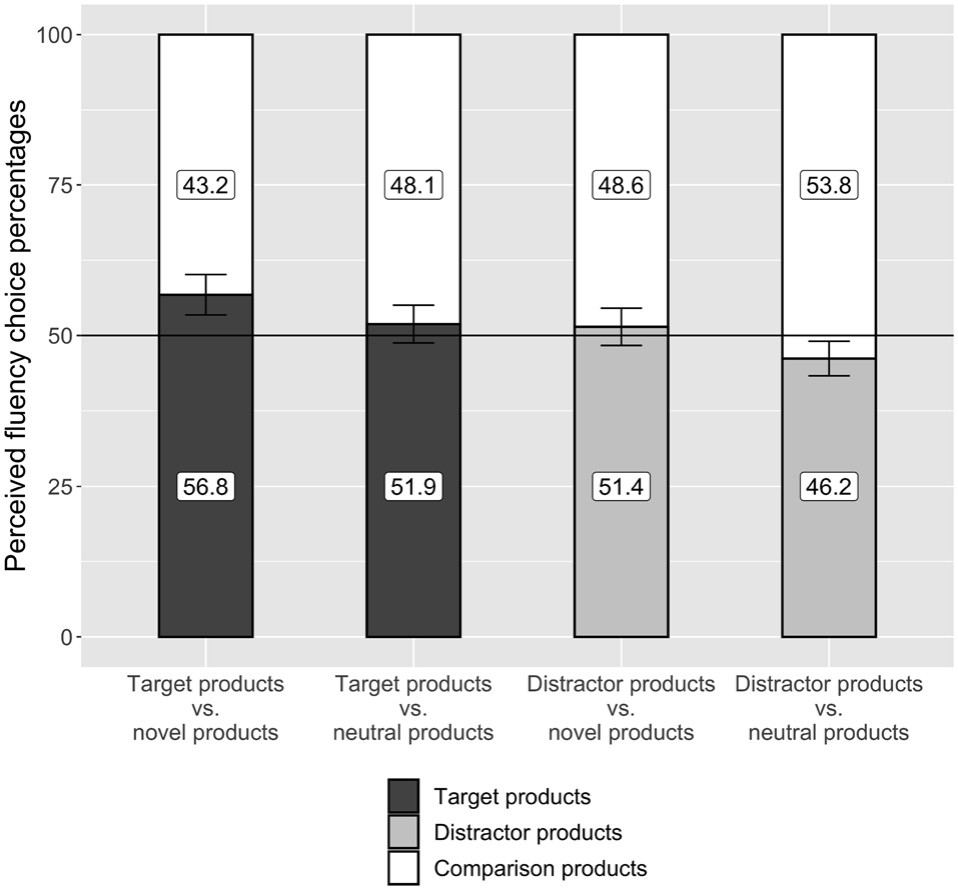
Perceived fluency choice percentages (Experiment 2). Perceived fluency choice percentages of the two-alternative forced-choice tasks in Experiment 2. Participants chose those choice options which they could perceive more fluently. Error bars show 95% confidence intervals.

Furthermore, we investigated in more detail whether target and distractor products are perceived more or less fluently than neutral and novel comparison alternatives. As expected, target products (*M* = 56.8%, *SD* = 18.2) were perceived more fluently than novel products during choice, *t*(115) = 3.99, *p* < 0.001, *d* = 0.37, BF_10_ = 152.90. However, target products (*M* = 51.9%, *SD* = 17.1) were not perceived more fluently than neutral products presented equally often and for an equally long duration, *t*(115) = 1.20, *p* = 0.234, BF_10_ = 0.21. Distractor products were perceived less fluently compared to neutral products during choice (*M* = 46.2%, *SD* = 15.6), *t*(115) = 2.64, *p* = 0.009, *d* = 0.25, BF_10_ = 2.81, but not perceived less fluently compared to novel products (*M* = 51.4%, *SD* = 16.8), *t*(115) = 0.92, *p* = 0.359, BF_10_ = 0.16. The results are displayed in Fig. 2.

## Discussion

Selective visual attention during search is a neuronal mechanism which helps people to identify what they are searching for and ignore the irrelevant^1,2^. For example, whenever people search for specific products during their grocery shopping, selective attention decreases the time necessary to find the products by increasing the neuronal selectivity for the target and inhibiting the neuronal responses for any distracting information. However, as indicated by previous research, search processes can also increase or decrease subsequent preferences for objects^12–14,19^. In the present research, we investigated whether the neuronal processes of facilitation and inhibition during search affect subsequent preferences during choice^12^. Specifically, we tested whether higher competition during search subsequently increases preferences for target products and reduces preferences for distractor products. Furthermore, we tested whether searching for target products, in the presence of distractor products, increases the perceived fluency for target products and decreases the perceived fluency for distracting products.

In accordance with previous research, Experiment 1 showed that former targets of a search can benefit in a subsequent preference choice task^12–14,19^. We found that searching for target products, in the presence of distractor products, increased the preferences for target products in comparison. Furthermore, target products were preferred over novel products subsequent to search. However, contrary to the predictions of Janiszewski et al.^12^, we found no negative effect of search on preferences for distractor products when compared to novel products. Instead, our results are in line with recent studies in which researchers did not observe a negative effect of search on subsequent distractor preferences, when the preference choice task was temporally separated from the search task^13,14,19^.

In the present research, we were specifically interested in whether mechanisms of biased competition during search can explain the effects of search on preferences. The biased competition model states that, when competition during search increases, the neuronal processing of relevant information and the inhibition of irrelevant information also increase^1,2^. Janiszewski et al.^12^ further assumed that an increase in competition during search also increases the effects of search on preference choices temporally separated from search. However, Experiment 1 provided no evidence that an increase in competition for visual processing during search can increase the effects of search on subsequent preferences. We found that target products were not preferred more often after highly competitive search, compared to less competitive search. Similarly, distractor products were not preferred less often after highly competitive search, compared to less competitive search. Therefore, our results question whether biased competition during search is indeed sufficient to explain the mechanism by which search affects subsequent preferences.

Experiment 1 also showed that, in line with previous research^12^, repeating a target during multiple consecutive search tasks increased the effect of search on target preferences. However, this repetition effect was limited to target products. For distractor products, we found no effect of repetition on preferences subsequent to search. The finding that distractors did not benefit from repeated exposure is interesting, because classical assumptions of the effects of mere exposure predict that repeated exposures alone can increase preferences^38^. The biased competition model offers one explanation why distractors might not benefit from mere exposure during search. During an ongoing search task, distractors are suppressed from further processing^1,20^. This suppression effect might also block mere exposure effects, but without necessarily devaluating preferences when choice is temporally separated from search^13,14,19^.

It is important to note that the evidence provided by Experiment 1 is in line with the general predictions of the biased competition model. We found that, for highly competitive search tasks, the response times to identify the target in consecutive search tasks diminished faster compared to less competitive search tasks. Similarly, and also predicted by the biased competition model, repeating a distractor product during subsequent highly competitive search tasks also decreased response times to identify the target products, compared to less competitive search. Our findings support that, in our design, higher competition during search indeed increased processes of facilitation and inhibition for targets and distractors, as predicted by the biased competition model. However, this neuronal modulation induced by biased competition during search apparently did not affect the preferences during subsequent choice.

Experiment 2 complemented Experiment 1 by testing whether search operations can influence subsequent preferences by affecting the perceived fluency to process the choice options. Janiszewski et al.^12^ initially assumed that search might affect the subsequent perception of target and distractor products, which can affect preferences during subsequent choice. Although the effects of search on perceived fluency had not been shown in previous research, researchers have provided extensive evidence that the subjectively perceived fluency to process choice options during the decision process can consistently affect a large variety of different outcome measures, like evaluations, liking ratings, or preferences^31–33^. Hence, fluency was a comprehensible candidate to explain the link between search and preferences. However, other researchers had formulated doubts on whether search processes can actually affect processing fluency^9,12,34^. The present research contributes to clarify this debate. Corresponding to the observed effects on preferences in Experiment 1, we found that searching for a target product, while ignoring distractor products, increased the perceived fluency for target products compared to novel products. Furthermore, and also corresponding to the effects on preferences in Experiment 1, we found no difference in the perceived processing fluency between distractors and novel products subsequent to search.

The results of Experiment 1 and 2 are in line with the assumption that search increases the fluency to perceive target products, compared to novel products, which subsequently increases preferences during choice. By contrast, the findings of Experiment 2 indicate that the fluency to perceive distractor products is not affected by preceding search. According to research on the effects of mere exposure, the mere presentation of stimuli should lead to increased fluency in the perception of these stimuli, and increased preferences^38^. However, this is not what we observed for distractor products. Hence, the results of our experiments suggest that effects usually produced by mere exposure are blocked for products which are distractors during search.

Furthermore, we found that distractor products during search are subsequently perceived less fluently compared to equally often, and equally long presented neutral products^12^. However, distractor products where not perceived more or less fluently compared to novel products. Hence, the effect between distractor and neutral products is likely to be driven by an increase in perceived fluency for the neutral products, not by a decrease in perceived fluency for the distractor products. A possible explanation for this difference in perceived processing fluency goes beyond inhibitory processes. Participants might have looked longer at the neutral products than at distractor products, and because of this attention advantage, participants might have perceived the neutral products more fluently than the distractor products in the subsequent task^13^.

While differences in visual attention durations might explain the differences in fluency between neutral and distractor products, it is also important to note that our results alone do not suffice to argue that search affects the fluency to perceive target products beyond mere exposure effects. Although we found that target products are perceived more fluently subsequent to search than novel products, we found no differences in the perceived fluency for target products and equally long, and equally often, presented neutral products. However, we would like to stress that, in other studies, researchers repeatedly observed effects of search on preferences for target products that cannot be explained by differences in exposure or visual attention^12–14,19^. For instance, Florack et al.^13^ used eye tracking to control for differences in visual attention during search and found that target products were preferred over neutral products even when target products and neutral products were attended for an equally long duration. We cannot say with reasonable certainty why we found no difference between the fluency to perceive target and neutral products subsequent to search in Experiment 2. Future research might investigate whether there is a difference in perceived fluency and preferences which we not considered in the present research.

We also like to point out that our results do not imply that, under different circumstances, distractor products cannot actually be devalued subsequent to search. Previous research showed that evaluations for stimuli can be lower when the evaluations takes place immediately after the search task^3,4^. However, in our design, we tested the effects of search on distractor products when the choice tasks were presented temporally separated from search, with a couple of minutes in between. It is possible that distractor devaluation effects take place immediately after the search tasks, but that these effects do not sustain for a longer duration. Researchers have observed such decays in effects of inhibition in related fields^39^.

In sum, we found that biased competition affects the processing of targets and distractors during search. We also found that search operations can affect the perceived fluency of objects subsequent to search, but that these effects are likely influenced by attention differences during search. Importantly, the effects of biased competition during search did not affect preferences during subsequent choices, and it is currently unclear by which mechanism search operations can affect subsequent preferences of objects. Therefore, it is important to explore alternative explanations to identify the mechanisms of search on preferences in future studies. As outlined above, differences in visual attention duration during search might affect the perceived fluency of objects presented during search tasks^13^. We recommend that future studies control for the actual differences in visual attention during search or measure the differences using eye tracking. Furthermore, future research might investigate whether forming and storing a mental representation of the target object in working memory, during the search process, affects how fluently the target is perceived subsequent to search^40–42^. In future studies, researchers might also investigate whether search can affect preferences by other fluency processes not related to visual perception fluency, for example, memory effects related to retrieval fluency^43^. Using different methods and developing new methodological approaches could also help to further investigate the validity and generalizability of the present findings and to further investigate the mechanisms by which search affects subsequent preferences.

## Conclusion

Our findings align with the basic assumptions of the biased competition model for ongoing search processes. When competition for visual processing during search was high, the processing of targets was facilitated and the inhibition of distractors was stronger compared to less competitive search. Our findings also indicate that the perceived fluency for targets can increase subsequent to search, although we found no evidence that this effect goes beyond mere exposure effects. Furthermore, our results indicate that mere exposure effects are blocked for distractors. Importantly, we found no indication for the assumption that biased competition during search is the mechanism responsible for the effects of search on preferences. Hence, we emphasize the necessity for future research to develop and investigate alternative explanations for how search affects preferences.

## Method

### Ethics statement

For all experiments, each participant gave informed consent upon arrival at the laboratory. We conducted all experiments in accordance with the Declaration of Helsinki^44^ and the local guidelines for studies with human participants of the Department of Occupational, Economic, and Social Psychology of the University of Vienna. The review board of the Department of Occupational, Economic, and Social Psychology of the University of Vienna approved that, for both experiments, participants’ rights and integrity were not threatened by our study designs (project numbers 2021/S/009 and 2021/S/012).

### Experiment 1

#### Design

We applied a 2 (attention: target product vs. distractor product) × 2 (competition: low vs. high) × 2 (repetition: one time vs. three times) within-subjects design.

#### Participants

An a priori simulation-based power analysis with the package SIMR^45^ for R^46^ with 1000 simulations revealed that at least 130 participants were necessary to detect the expected main effects with a power of at least 80%. We recruited 174 psychology students at the University of Vienna in exchange for course credit (*M*_*age*_ = 21.0 years, *SD*_*age*_ = 3.2 years; 134 women, 40 men).

#### Material

We used 168 products from seven different product categories (chips, energy drinks, strawberry jam, ketchup, lemonade, soda, sparkling water) from online stores of foreign supermarkets. We randomized the assignment of products to the experimental conditions and the order of the product categories for each participant. Each participant saw all products.

#### Stimulus presentation

We presented the study on 24-inch monitors with a resolution of 1920 pixels width and 1080 pixels height at 60–70 cm in front of the participants. The maximum size of the products presented on screen was limited by 250 pixels width and 500 pixels height. For each product exposed during search, we also reduced the visibility during search to 65% (35% alpha transparency) so that two products with an overlay could still be distinguished from each other. When two products were presented together during search, their centers were separated by 700 pixels horizontally (low competition, no overlay) or by only 60 pixels (high competition, with overlay).

#### Procedure

Participants declared consent, were briefed on the study procedure, and completed training on the procedure. In the experiment, we presented seven exposure phases each followed by a preference choice phase. In the exposure phase, we presented target and distractor products, and asked participants to select the location of the target product. After each exposure phase, we presented a short comic strip for 10 s. The purpose of these comics was to separate the exposure phase from the following preference choice phase. In each preference choice phase, participants indicated in two-alternative forced-choice tasks which product they preferred. The exposure phase and the choice tasks are described below. After participants completed all exposure and choice phases, they answered some basic demographic questions.

#### Exposure phase

**Figure 3:**
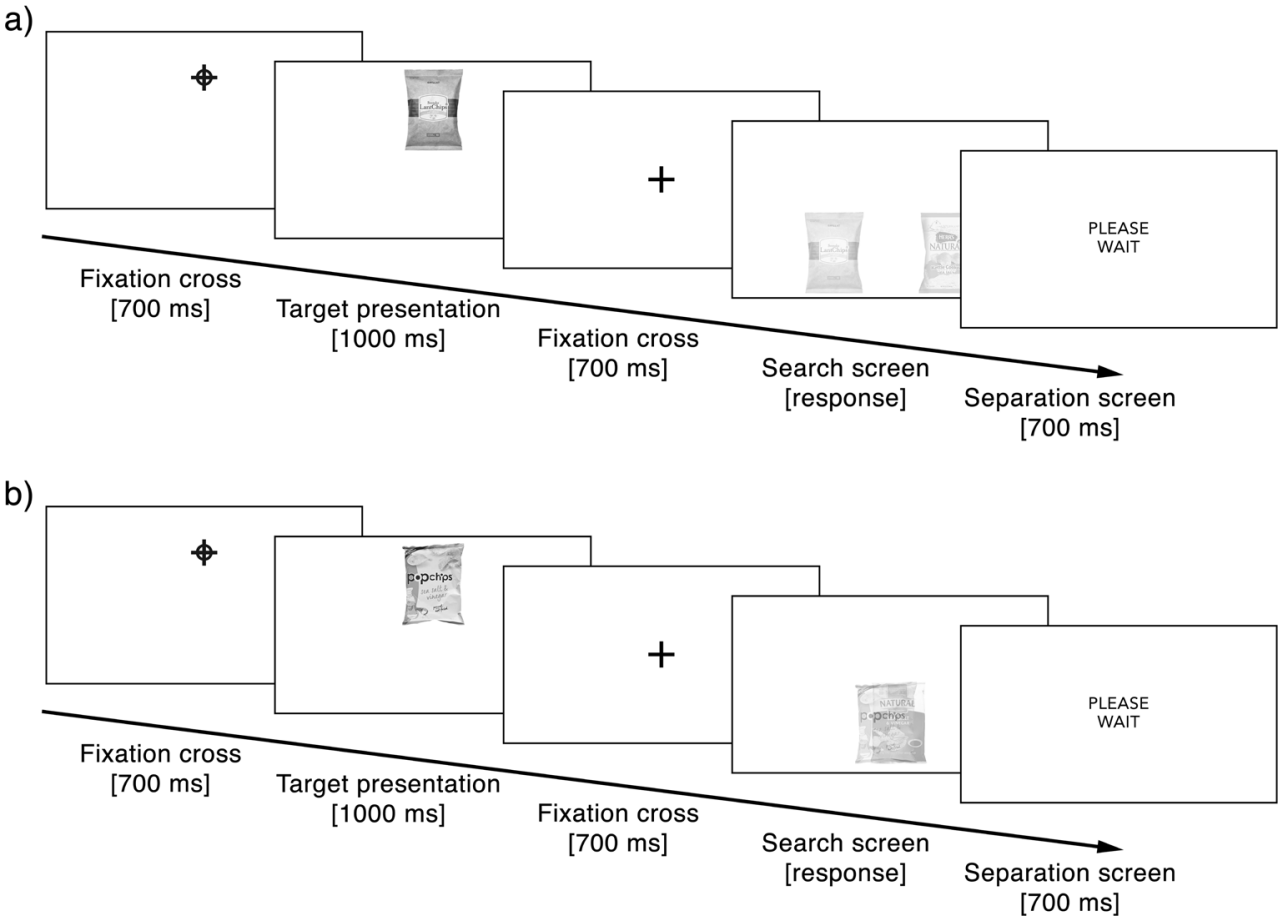
Exposure phase (Experiment 1). Example sequence of one search task during the exposure phase in Experiment 1 for (**a**) low competitive search and (**b**) highly competitive search.

Participants searched for products in seven separate exposure phases, one for each product category. Each exposure phase contained six search trials. Each of these trials was high or low in competition. Furthermore, in each search trial, either only the target or only the distractor was repeated in three consecutive search tasks, or either the target or the distractor was presented in only one search task. Figure 3 illustrates the procedure for one search task.

Each search task followed the same procedure. First, a blue fixation cross surrounded by a blue circle, central in the upper half of the screen, indicated that a target product would follow (700 ms). Participants then saw a target product with full visibility at the location where the fixation cross had appeared (1000 ms). After a black fixation cross in the center of the screen (700 ms), two products were simultaneously exposed in the bottom half of the screen with reduced visibility. Both products were either exposed separated from each other, or with an overlay, until participants indicated the location of the target product by pressing “A” (more on the left side) or “L” (more on the right side) on the keyboard. The target was randomly presented on the left or right. After participants pressed the corresponding key, a separation screen appeared with the text “Please wait” to prepare participants for the next trial (700 ms). When the side of the target was not correctly indicated, an error message (“This was wrong!”) was presented after the separation screen (1000 ms).

#### Preference choice phase

In each of the seven choice phases, each participant made eight preference decisions in two-alternative forced-choice tasks. We presented for each combination of competition and repetition search trials one former target product and one former distractor product form the exposure phase. Each target and distractor product was compared to a novel product not exposed before choice.

The procedure was the same for each trial. Following a black fixation cross in the center of the screen (700 ms), participants saw the two choice options with full visibility (no alpha transparency) side-by-side on the screen for a limited time (1000 ms). Which product was presented on the left or right side was randomized per trial. After the screen was cleared, a choice screen appeared with no time limit. The participants had to indicate the location of the product they preferred by pressing “A” (left product) or “L” (right product) on the keyboard. After participants made their choice, a separation screen labeled “Please wait” appeared (700 ms), before the next choice task started.

### Experiment 2

#### Design

We applied a 2 (attention: target product vs. distractor product) × 2 (comparison: neutral product vs. novel product) within-subjects design.

#### Participants

We calculated an a priori power analysis with G*Power^47^. We expected an effect size of *d* = 0.25 and aimed for a power of 0.80 at an alpha level of 0.05 in a one-sample *t*-test to detect a significant difference from choosing target products over neutral or novel products during the fluency perception choice tasks. This analysis resulted in a necessary sample of at least 101 participants. We recruited 116 psychology students at the University of Vienna in exchange for course credit (*M*_*age*_ = 21.5 years, *SD*_*age*_ = 3.0 years; 82 women, 33 men, no information on age and gender for one participant).

#### Material

We used 80 products from eight different product categories (chips, cookies, energy drinks, gums, strawberry jam, ketchup, lemonade, sparkling water) from online stores of foreign supermarkets. We randomized the assignment of products to the experimental conditions and the order of the product categories for each participant. Each participant saw all products.

#### Procedure

Participants declared consent, were briefed on the study procedure, and completed training on the procedure. In an exposure phase, we presented target, distractor, and neutral products, and asked participants to select the location of the target product. After the exposure phase, we consecutively presented six comic strips of 10 s each. The purpose of these comics was to separate the exposure phase from the subsequent perceived fluency choice tasks. In these choice tasks, participants indicated in two-alternative forced-choice tasks which product they perceived more fluently. The search tasks of the exposure phase and the subsequent fluency choice tasks are described in detail below. After participants completed the fluency choice tasks, they answered some basic demographic questions.

#### Exposure phase

**Figure 4:**
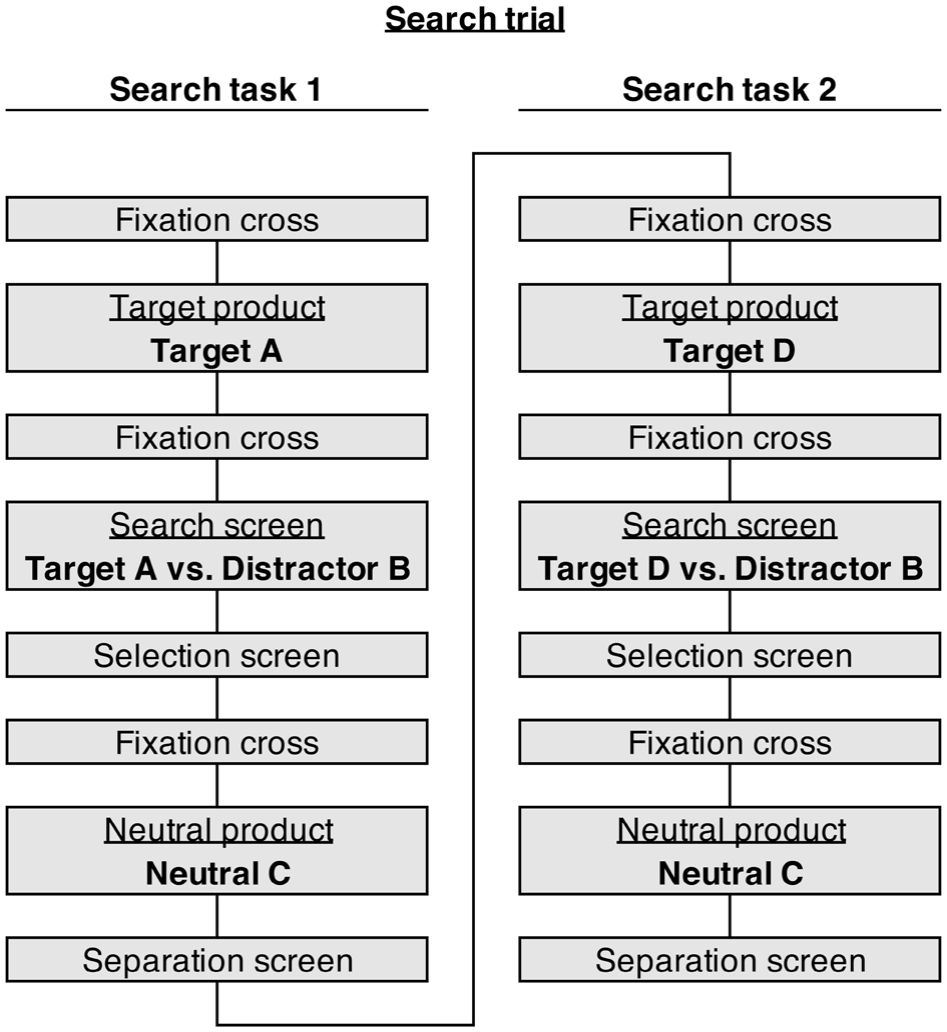
Trial structure (Experiment 2). Conceptual overview of the trial structure of one search trial in the exposure phase of Experiment 2. One trial consists of two search tasks (Search Task 1 and Search Task 2).

**Figure 5:**
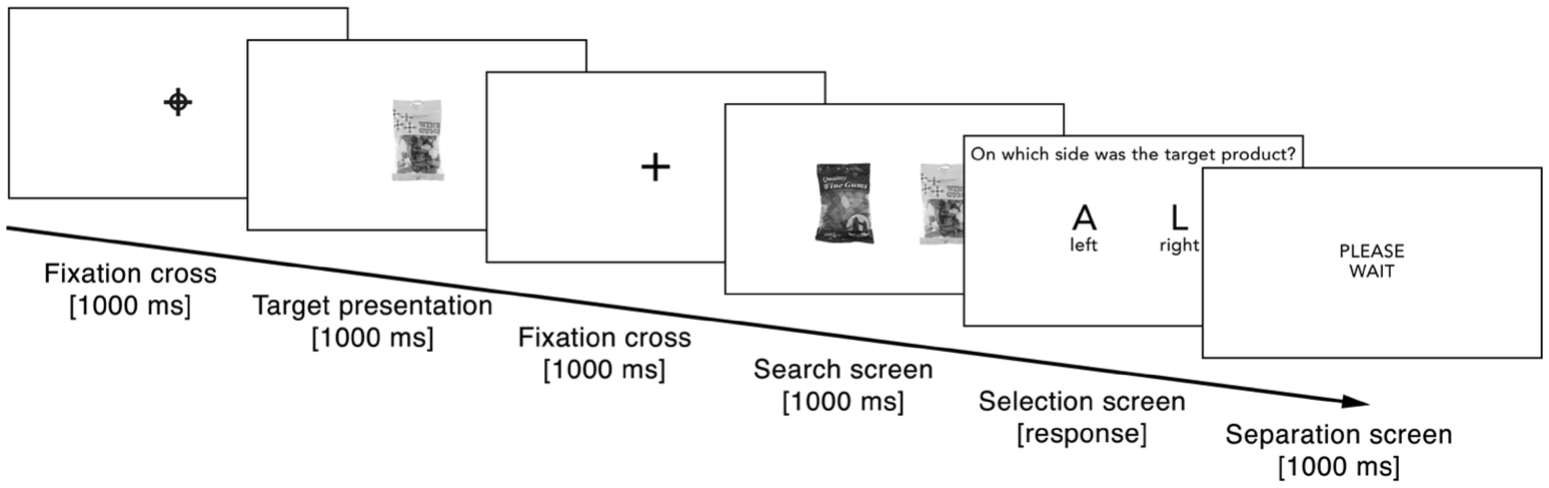
Exposure phase (Experiment 2). Example sequence of one search task during the exposure phase in Experiment 2.

Each participant completed a total of 16 search trials in the exposure phase. Each of these search trials consisted of a combination of two search tasks (see Fig. 4). The procedure for all search trials in the exposure phase was the same. We applied this design to keep the duration and frequency of the presentation of target, distractor, and neutral products constant. In each trial, we presented every product twice for 1000 ms. In the first search task of the trial, we presented the target product twice for 1000 ms, and the distractor and neutral products each once for 1000 ms. In the second search task of the trial, we presented the same distractor and neutral product again for 1000 ms, but with a new target product. Figure 5 illustrates the procedure of the search tasks.

At the beginning of each search task, a blue fixation cross surrounded by a blue circle, placed in the middle of the screen, indicated that a target product would follow (1000 ms). Participants were then presented with a target product in the middle of the screen where the fixation cross had appeared (1000 ms). After a black fixation cross in the middle of the screen (1000 ms), we presented two products simultaneously on the left or right screen side, one being the target product and the other a distractor product (1000 ms). The target was randomly presented on the left or right. On the subsequent screen participants had to indicate the side on which the target product had appeared by pressing “A” (left side) or “L” (right side) on the keyboard. The target was randomly presented on the left or right.

After participants pressed the corresponding key, a black fixation cross appeared in the middle of the screen (1000 ms), before the neutral product was presented in the middle of the screen (1000 ms). We told participants that the purpose of this screen was to clear their visual memory, but, in truth, this product represented the neutral comparison alternative used in the subsequent choice phase. Finally, a separation screen appeared with the text “Please wait” to prepare participants for the next trial (1000 ms). When the side of the target was not correctly indicated, an error message (“This was wrong!”) appeared after the separation screen (1000 ms).

#### Perceived fluency choice task

Each participant made 32 two-alternative forced choices in which the task was choosing those products which could be perceived more easily. Participants made eight choices for each of the following comparison conditions: target products compared to neutral products, target products compared to novel products, distractor products compared to neutral products, and distractor products compared to novel products. Half of the distractor products were randomly compared against a neutral product and the other half against a novel product of the same category. For the target products, from each search trial, we randomly selected one of the two products presented as target products and randomly compared half of them against neutral products and the other half against novel products.

The procedure was the same for each trial. Following a black fixation cross in the middle of the screen (1000 ms), participants saw the two choice options for a limited time together on the screen (1000 ms). Which product was presented on the left or right side was randomized per trial. After the screen was cleared, a choice screen appeared with no time limit. The participants had to indicate the product they perceived more fluently (“Which product did you perceive more fluently?”) by pressing “A” (left product) or “L” (right product). After participants made their choice, the separation screen “Please wait” appeared before the next choice trial started (1000 ms).

## Supplementary Information

Supplementary Information.

## Data Availability

Data sets and R scripts used for data analyses are available for download (https://osf.io/mysz8). Please note that, due to data protection, we did not include demographic information of participants in these files.
