# Supplementary material for: Investigating the mechanisms by which selective attention affects subsequent preferences and choice
Source: Sci Rep. 2022 Nov 11;12:19345. doi: 10.1038/s41598-022-23859-6 (PMC9652248; doi:10.1038/s41598-022-23859-6)
Supplement: Supplementary file 1 — Supplementary Information. [file 41598_2022_23859_MOESM1_ESM.pdf]

Investigating the mechanisms by which selective attention affects subsequent preferences and choice

Martin Egger and Arnd Florack

University of Vienna, Austria

Supplementary Analyses

Experiment 1

Effect of processing speed on product preferences

We analyzed whether the increase in processing speed during search, found when target or distractors repeated during multiple search tasks, can predict preferences during choice. We calculated generalized linear mixed-effects models (GLMM) with the preference choice as binary outcome variable and the increase in processing speed as continuous fixed factor predictor. Again, we computed the increase in processing speed as the regression coefficients of the three response times for each repetition trial. We z-standardized the continuous predictor to prevent scaling issues in the model estimation.

Our analysis revealed that the increase in processing speed for target products during search could not predict preference choices, neither for low competitive search, $b = <0.01$, $SE = 0.06$, $z = 0.03$, $p = .977$, 95% CI [-0.12, 0.13], nor for highly competitive search, $b = 0.05$, $SE = 0.06$, $z = 0.80$, $p = .424$, 95% CI [-0.07, 0.17]. Similarly, the increase in processing speed for distractor products during search could not predict preference choices, neither for low competitive search, $b > -0.01$, $SE = 0.06$, $z = 0.06$, $p = .956$, 95% CI [-0.13, 0.11], nor for highly competitive search, $b = 0.04$, $SE = 0.06$, $z = 0.62$, $p = .539$, 95% CI [-0.9, 0.16].

Effect of total presentation time on product preferences

As an additional analysis, we controlled for mere exposure effects on preferences, since the presentation durations of targets and distractors in the search tasks varied due to the response-dependent exposure duration. Specifically, we tested whether the total duration of product presentation during search had an effect on preferences. To avoid confounds, due to the different number of presentations, we calculated separate GLMM for one and three repetition trials. We included the total duration of product presentation during search tasks for each participant as a continuous fixed factor in the model. The preference choice was the binary outcome. We *z*-standardized the continuous fixed factor predictor to prevent scaling issues in the model estimation.

Our analysis revealed that the total time of target presentation during search did not predict preferences during choice, neither for one search task, $b = 0.05$, $SE = 0.04$, $z = 1.10$, $p = .270$, 95% CI [-0.03, 0.13], nor for three subsequent search tasks, $b = 0.04$, $SE = 0.04$, $z = 0.95$, $p = .342$, 95% CI [-0.04, 0.13]. Similarly, the total time of distractor presentation during search did not predict preferences during choice, neither for one search task, $b = -0.03$, $SE = 0.04$, $z = 0.84$, $p = .399$, 95% CI [-0.11, 0.05], nor for three subsequent search tasks, $b = -0.01$, $SE = 0.04$, $z = 2.08$, $p = .835$, 95% CI [-0.09, 0.07].
